# Depression and loneliness among parents of premature infants admitted to Neonatal Intensive Care Unit (NICU)

**DOI:** 10.1192/j.eurpsy.2023.386

**Published:** 2023-07-19

**Authors:** S. Kouri, D. Briana, I. Koutelekos, A. Zartaloudi

**Affiliations:** ^1^ National and Kapodistrian University of Athens; ^2^University of West Attica, Athens, Greece

## Abstract

**Introduction:**

When newborns are born prematurely, it is often necessary to be hospitalized in a Neonatal Intensive Care Unit (NICU). As a result, they are immediately separated from both parents, who experience an intense emotional burden throughout their baby’s hospitalization. Newborns’ entrance in Intensive Care Unit can trigger negative emotions in both parents.

**Objectives:**

To assess the feeling of loneliness, depressive symptoms and post-traumatic stress of parents with premature infants who are hospitalized in Neonatal Intensive Care Units (NICUs).

**Methods:**

Our sample consisted of 251 parents, whose newborn was hospitalized in the Neonatal Intensive Care Unit (NICU) of three hospitals in Athens, the capital of Greece. The data were collected through a questionnaire which included (a) questions related to socio-demographic and clinical data, (b) the Center for Epidemiological Studies-depression scale, (c) the UCLA Loneliness Scale (d) ) the Impact of Event Scale- Revised- Greek version (IES-R-Gr) for the detection of post-traumatic stress.

**Results:**

The majority of our sample were women (69.7%) with a mean age of 32.2 years (SD = 15.4 years). Mothers experienced significantly higher scores on each scale, suggesting more symptoms of depression and post-traumatic stress as well as a higher sense of loneliness compared to fathers. Parents whose infants were underweight and parents with previous experience of hospitalization in NICU exhibited a statistically significant higher sense of loneliness. 62.6% of parents developed depressive symptoms. There was a statistically significant positive correlation between feeling lonely and the onset of depressive symptoms and a statistically significant negative correlation between psychological support from hospital staff and the appearance of depressive symptomatology. The 60.1% of our participants showed symptoms of post-traumatic stress. The more depressive symptoms the participants exhibited, the more symptoms of avoidance, resuscitation, and overstimulation were noted. The greater the feeling of loneliness, the more symptoms of avoidance the participants exhibited.

**Conclusions:**

Early detection of depressive symptoms, loneliness and post-traumatic stress in parents whose newborn is hospitalized in NICU is of major importance. Consequently, a systematic and well-organized training of the staff working in NICU should be provided. Specific protocols, as well as individualized interventions should be implemented to manage the needs and feelings of this vulnerable population.

**Disclosure of Interest:**

None Declared

